# Glucose dysregulation and subclinical cardiac dysfunction in older adults: The Cardiovascular Health Study

**DOI:** 10.1186/s12933-022-01547-z

**Published:** 2022-06-20

**Authors:** Parveen K. Garg, Mary L. Biggs, Jorge R. Kizer, Sanjiv J. Shah, Bruce Psaty, Mercedes Carnethon, John S. Gottdiener, David Siscovick, Kenneth J. Mukamal

**Affiliations:** 1 Division of Cardiology, University of Southern California Keck School of Medicine, 1510 San Pablo St. Suite 322, Los Angeles, CA 90033 USA; 2 Department of Biostatistics, University of Washington, Seattle, WA USA; 3 Cardiology Section, San Francisco Veterans Affairs Health Care System, and Department of Medicine, University of California San Francisco, San Francisco, CA USA; 4 Department of Epidemiology and Biostatistics, University of California San Francisco, San Francisco, CA USA; 5 Division of Cardiology, Northwestern University Feinberg School of Medicine, Chicago, IL USA; 6 Cardiovascular Health Research Unit, Departments of Medicine, Epidemiology and Health Services, University of Washington, Seattle, WA USA; 7 Department of Preventive Medicine, Northwestern University Feinberg School of Medicine, Chicago, IL USA; 8 Department of Medicine, University of Maryland School of Medicine, Baltimore, MD USA; 9 New York Academy of Medicine, New York, NY USA; 10 Department of Medicine, Beth Israel Deaconess Medical Center, Boston, MA USA

**Keywords:** Glucose dysregulation, Insulin resistance, Cardiac strain, Heart failure

## Abstract

**Objective:**

We evaluated whether measures of glucose dysregulation are associated with subclinical cardiac dysfunction, as assessed by speckle-tracking echocardiography, in an older population.

**Methods:**

Participants were men and women in the Cardiovascular Health Study, age 65+ years and without coronary heart disease, atrial fibrillation, or heart failure at baseline. We evaluated fasting insulin resistance (IR) with the homeostatic model of insulin resistance (HOMA-IR) and estimated the Matsuda insulin sensitivity index (ISI) and insulin secretion with an oral glucose tolerance test. Systolic and diastolic cardiac mechanics were measured with speckle-tracking analysis of echocardiograms. Multi-variable adjusted linear regression models were used to investigate associations of insulin measures and cardiac mechanics.

**Results:**

Mean age for the 2433 included participants was 72.0 years, 33.6% were male, and 3.7% were black. After adjustment for age, sex, race, site, speckle-tracking analyst, echo image and quality score, higher HOMA-IR, lower Matsuda ISI, and higher insulin secretion were each associated with worse left ventricular (LV) longitudinal strain and LV early diastolic strain rate (p-value < 0.005); however, associations were significantly attenuated after adjustment for waist circumference, with the exception of Matsuda ISI and LV longitudinal strain (increase in strain per standard deviation increment in Matsuda ISI = 0.18; 95% confidence interval = 0.03–0.33).

**Conclusion:**

In this cross-sectional study of older adults, associations of glucose dysregulation with subclinical cardiac dysfunction were largely attenuated after adjusting for central adiposity.

**Supplementary Information:**

The online version contains supplementary material available at 10.1186/s12933-022-01547-z.

## Introduction

Glycemic dysregulation and heart failure (HF) are chronic diseases with substantial morbidity and mortality and are increasing in prevalence [1, 2]. Both disorders are closely tied to aging, with their highest burden seen among older adults. Experimental and clinical studies have demonstrated that abnormal glucose metabolism is implicated in the pathogenesis of cardiovascular disease, including HF [3].

The association between glycemic dysregulation and HF has been long described [4]. The postulated biological pathways include small vessel disease, increase in oxidative stress, and increased collagen-bound advanced-glycation end products that promote ventricular hypertrophy by increasing myocardial collagen deposition and fibrosis [3, 5, 6]. Interestingly, obesity and insulin resistance have been more closely linked to incidence of heart failure with preserved ejection fraction (HFpEF) than with heart failure with reduced ejection fraction (HFrEF), especially among women [7].

The pathophysiology of glycemic dysregulation entails development of hepatic insulin resistance, which eventuates in fasting glucose elevations, and skeletal muscle insulin resistance and pancreatic beta-cell failure, which leads to post-prandial or post-load hyperglycemia [8]. Among older adults, post-load hyperglycemia is the predominant contributor to abnormal glucose homeostasis [9]. Prior work in CHS has shown that post-load glucose is more strongly associated than fasting glucose with incidence of CVD [10, 11].

The development of speckle-tracking echocardiography allows earlier evaluation of impaired myocardial function than previously possible with traditional echocardiographic measures [12]. Assessment of candidate risk factors’ impact on myocardial mechanics can provide insights into pathophysiology and potentially open new paths to prevention of myocardial disease, heart failure and their consequences. Indeed, previous studies have linked abnormal glucose homeostasis to impaired myocardial mechanics [13–16]. The differential impact of post-load and fasting glucose on myocardial deformation has not been studied.

In an older population without prevalent cardiovascular disease, we determined whether a wide variety of glucose measures, including fasting glucose, 2-h glucose, homeostatic model of insulin resistance (HOMA-IR), Matsuda insulin sensitivity index (ISI) and insulin secretion, are associated with worse LV systolic strain (LVLS), LV early diastolic strain rate (LVDSR), and LA reservoir strain (LARS), as measured by speckle-tracking echocardiography.

## Materials and methods

### Study participants

**Fig. 1:**
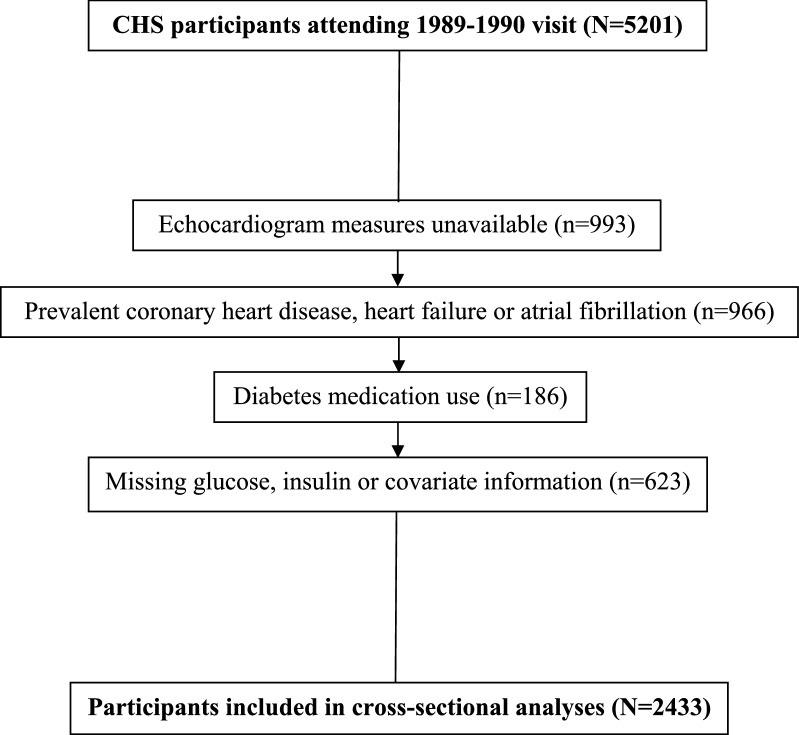
Flowchart of participants included in the analysis

The Cardiovascular Health Study (CHS) includes 5201 original participants recruited in 1989–1990; an additional 687 participants were recruited in 1992–1993 but underwent a more limited baseline examination that precluded inclusion in these analyses. Study participants were randomly selected from Medicare-eligibility lists in four US communities (Forsyth County, NC; Washington County, MD; Sacramento County, CA; and Pittsburgh, PA). Detailed methodology and design of the CHS has been reported previously [17]. The study was approved by the institutional review board at each center, and informed consent signed by all participants was obtained. Figure 1 shows the flowchart of participants for this study. There were 2433 participants eligible for this analysis.

### Echocardiography—cardiac mechanics

The design of the echocardiography protocol used in CHS has been described in detail elsewhere [18]. Briefly, participants underwent routine transthoracic echocardiography at baseline. Images were recorded onto super-VHS tape during acquisition with Toshiba SSH-160A cardiac ultrasound machines. A standardized protocol was used to obtain 2D, guided M-mode, spectral and color flow Doppler data. Videotapes were sent to a central echocardiography core laboratory (Irvine, CA for the 1989–1990 echocardiograms) where images were displayed and digitized. Measurements were made from digitized images using an off-line image-analysis system equipped with customized computer algorithms.

From 2016 to 2018, archived CHS echocardiograms were digitized using the TIMS 2000 DICOM system (Foresight Imaging, Chelmsford, MA), using methods developed by our group for a similar analysis done in the Hypertension Genetic Epidemiology (HyperGEN) Study [19]. Cine loops of 2–4 cardiac cycles from the apical 4-chamber view were digitized at a frame rate of 30 frames per second and stored offline in DICOM format (Northwestern University; Chicago, IL). Speckle-tracking echocardiography was subsequently performed for strain analysis using specific software (TOMTEC Cardiac Performance Analysis, v4.5, Unterschleiβheim, Germany) by 5 experienced readers. All echocardiograms were assigned chamber-specific image quality scores (0–4) based on degree of visualization of the myocardium and cardiac structures, as described previously [19]. Speckle-tracking measurements were made with the R-R wave ECG gating to define the cardiac cycle. All strain measurements were made in the apical 4-chamber view only based on our prior findings in HyperGEN indicating similar values of speckle-tracking strain measures in the apical 4-, 3-, and 2-chamber view [19]. The LV endocardial border was traced manually in the apical 4-chamber view for creation of the LVLS curve. Six segments of the LV were identified by strain software; segments which did not track appropriately were removed from the analysis, and the average of the remaining segments was generated, from which LVLS and LVDSR were derived. LARS was measured using similar methods after manually tracing the LA endocardial border in the apical 4-chamber view. Given that the ventricular cycle was the reference point, all LA strain values were reported as positive absolute percentages. LARS was defined as the peak average LA strain and corresponded to ventricular systole.

### Insulin and glucose measures

Serum samples were obtained at baseline (1989–1990) after an overnight fast of at least 8 h, and again 2 h after a 75-g oral glucose challenge. Insulin was measured with a competitive radioimmunoassay (Diagnostic Products Corporation), and glucose was measured with an enzymatic method [20].

The homeostatic model of insulin resistance (HOMA-IR) is a measure of fasting IR calculated using the following formula: [fasting glucose (mmol/l)]*[fasting insulin (U/ml)]/22.5 [21]. The Matsuda insulin sensitivity index (Matsuda ISI) is a measure of fasting and post-glucose loading IR and is calculated as insulin sensitivity = $\sqrt {\left( {{{10,000} \mathord{\left/ {\vphantom {{10,000} {\left( {{\text{G}}_{0} *{\text{I}}_{{0}} *{\text{G}}_{120} *{\text{I}}_{{{120}}} } \right)}}} \right. \kern-\nulldelimiterspace} {\left( {{\text{G}}_{0} *{\text{I}}_{{0}} *{\text{G}}_{120} *{\text{I}}_{{{120}}} } \right)}}} \right)}$, where G_0_ is glucose concentration (mg/dl) at time 0, I_0_ is the insulin concentration at time 0 (mmol/ml), G_120_ is the glucose concentration at time 120 min, and I_120_ is the insulin concentration 120 min obtained from an OGTT [22]. Fasting and post-load insulin secretion was measured using the second phase of the Stumvoll index [23].

### Covariates

Age, gender, race, smoking status, and alcohol consumption, and physical activity were obtained by self-report. Recent medication use was assessed using a medication inventory. Smoking status was categorized as current, former, and never use. Alcohol consumption referred to number of alcoholic drinks consumed per week. Physical activity levels referred to the energy in kilocalories expended in weekly household and leisure-time physical activity estimated from the Minnesota Leisure Time Activities Questionnaire.

Waist circumference was measured at the level of the umbilicus. Hypertension was defined as: (1) systolic blood pressure ≥ 140 mmHg, diastolic ≥ 90 mmHg, or (2) self-report of physician-diagnosed hypertension accompanied by use of medications for hypertension. Serum low-density lipoprotein cholesterol (LDL-C) and high-density lipoprotein cholesterol (HDL-C) were measured by enzymatic methods. Cystatin C was measured by means of a particle-enhanced immunonephelometric assay (N Latex Cystatin C; Dade Behring) with a nephelometer (BNII; Dade Behring). Glomerular filtration rate was estimated with the use of the CKD-EPI cystatin C equation (eGFRcys) without demographic coefficient.

### Statistical analysis

Descriptive statistics (mean and standard deviation for continuous variables and counts and percentages for categorical variables) were used to summarize baseline characteristics of participants stratified by quartiles of fasting glucose and 2-h glucose. Linear pairwise correlations amongst insulin and glucose measures (fasting glucose, 2-h glucose, HOMA-IR, Matsuda ISI, and Stumvoll index) were estimated using Pearson’s correlation coefficients. Multi-variable adjusted linear regression models were used to investigate the associations of fasting glucose (n = 2426), 2-h glucose (n = 2345), HOMA-IR (n = 2426), Matsuda ISI (n = 2336), and Stumvoll index (n = 2336) with absolute values (higher values indicating better mechanics) of: (1) LVLS, (2) LVDSR, and (3) LARS. Regression (β) coefficients per 1 SD increment for each glucose and insulin measure are presented as the primary metric of association. Analyses were adjusted for potential confounders and included age, race, sex, clinic site, waist circumference, cigarette smoking, systolic blood pressure, anti-hypertensive therapy, alcohol, LDL-C, HDL-C, eGFR_cys_, heart rate, speckle-tracking analyst, and image quality. Waist circumference was chosen because prior CHS work has demonstrated it to be more strongly associated with cardiac strain than was body-mass index [24].

To determine which covariates were most important as potential confounders, we tested individually the impact of each of the model 2 covariates in separate regression models. We repeated this process after further including waist circumference as a model 1 covariate.

Finally, given the previously known association between waist circumference and worse cardiac strain, we also determined whether glucose and insulin measures may have mediated this association, using a methodological approach that accounts for mediation and interaction and that has been previously used in this cohort [25].

## Results

The mean age for included participants was 72.0 years, 33.6% were male, and 3.7% were black. Table 1 reports participant characteristics across quartiles of fasting glucose. Compared to participants in the lowest quartile of fasting glucose, those in higher quartiles were more likely to be male, be black, have hypertension, be on anti-hypertensive medications, drink alcohol, and have a smoking history. Baseline waist circumference, LDL-c, and SBP were higher while physical activity, HDL-c and eGFR_cys_ were lower for participants in higher quartiles of fasting glucose. Additional file 1: Table S1 reports participant characteristics across quartiles of 2-h glucose. Associations were similar except that compared to participants in the lowest quartile of 2-h glucose, those in higher quartiles were less likely to drink alcohol or have a smoking history.

**Table 1:** Baseline characteristics of CHS participants according to fasting glucose quartiles (n = 2433)

Characteristic	Fasting glucose quartile				*p*-value
	≤ 93	> 93–99	> 99–107	> 107	
Age, years	71.83 ± 5.06	72.09 ± 5.27	72.47 ± 5.29	71.70 ± 5.02	0.99
Waist circumference, cm	86.84 ± 12.21	90.52 ± 12.21	93.04 ± 12.25	97.82 ± 12.18	< 0.01
Body mass index, kg/m^2^	24.26 ± 3.75	25.64 ± 4.01	26.41 ± 4.36	28.19 ± 4.33	< 0.01
Heart rate	33.12 ± 4.60	33.42 ± 4.98	33.45 ± 5.13	34.56 ± 5.75	< 0.01
Physical activity, kcal/week	2110.35 ± 2231.61	1942.79 ± 2147.84	1699.99 ± 1800.06	1897.52 ± 2320.83	0.02
Systolic BP, mmHg	131.09 ± 20.74	132.99 ± 20.73	137.61 ± 20.98	137.47 ± 20.43	< 0.01
LDL, mg/dl	128.96 ± 34.96	131.67 ± 35.41	132.66 ± 33.48	134.59 ± 37.74	< 0.01
HDL, mg/dl	62.19 ± 16.40	57.65 ± 15.94	56.18 ± 14.83	51.13 ± 14.09	< 0.01
eGFR-cys_c_	82.31 ± 18.75	80.45 ± 18.16	78.48 ± 18.87	77.49 ± 18.52	< 0.01
Male	131 (20.3%)	214 (35.1%)	211 (36.7%)	262 (43.3%)	< 0.01
Black	23 (3.6%)	21 (3.4%)	15 (2.6%)	31 (5.1%)	0.26
Hypertension	178 (27.6%)	209 (34.4%)	240 (41.7%)	289 (47.8%)	< 0.01
Stroke	13 (2.0%)	15 (2.5%)	19 (3.3%)	16 (2.6%)	0.34
Anti-hypertensive use	166 (25.8%)	200 (32.8%)	224 (39.0%)	285 (47.1%)	< 0.01
Smoking status					0.03
Never	345 (53.6%)	276 (45.3%)	277 (48.2%)	271 (44.8%)	
Former	228 (35.4%)	252 (41.4%)	237 (41.2%)	252 (41.7%)	
Current	71 (11.0%)	81 (13.3%)	61 (10.6%)	82 (13.6%)	
Alcohol use, drinks/wk					0.10
None	299 (46.4%)	272 (44.7%)	251 (43.7%)	284 (46.9%)	
1–7	275 (42.7%)	248 (40.7%)	230 (40.0%)	227 (37.5%)	
> 7	70 (10.9%)	89 (14.6%)	94 (16.3%)	94 (15.5%)	

Continuous variables are expressed as mean (SD). Categorical variables are N (%)

*LDL* low-density lipoprotein; *HDL* high-density lipoprotein, *eGFR*_*cys*_ estimated glomerular filtration rate cystatin C

Correlations among glucose and insulin measures are reported in Table 2. Strongest observed correlations were between fasting glucose and 2-h glucose (r = 0.664) and Matsuda ISI and 2-h glucose (r = − 0.566).

**Table 2:** Correlation matrix of glucose and insulin measures (n = 2343)

	Fasting glucose	2-h glucose	HOMA-IR	Matsuda ISI	Stumvoll index
Fasting glucose					
2-h glucose	0.664				
HOMA-IR	0.493		0.278		
Matsuda ISI	− 0.359		− 0.566	− 0.349	
Stumvoll index	− 0.253		− 0.383	0.335	− 0.275

Values represent Pearson’s correlation coefficients. p < 0.001 for all correlations

*HOMA-IR* homeostatic model of insulin resistance, *ISI* insulin sensitivity index

Table 3 shows the associations of glucose and insulin measures, all on a per-SD scale, with three measures of strain. After adjusting for age, sex, race, clinic site, reader, and image quality, a higher fasting glucose, 2-h glucose, HOMA-IR, and Stumvoll index were each associated with worse LVLS and LVDSR while a higher Matsuda ISI was associated with better LVLS and LVDSR. Fasting glucose and the Matsuda ISI were the only measures associated with LARS. Except for Matsuda ISI and LV longitudinal strain (beta coefficient per standard deviation increment in Matsuda ISI = 0.18; 95% confidence interval = 0.03–0.33), all associations were meaningfully attenuated after additional adjustment for waist circumference, cigarette smoking, systolic blood pressure, anti-hypertensive therapy, alcohol, LDL-C, HDL-C, eGFR_cys_, and heart rate (Table 3). In analyses evaluating the impact of each model 2 covariate (Table 4), adjustment for waist circumference had the largest impact on the associations of glucose and insulin measures with all cardiac strain measures. In essentially every instance, the regression coefficient with adjustment for waist circumference was closer to the null than with adjustment for any other model 2 covariate. When waist circumference was then included as part of Model 1, additional adjustment for heart rate had the largest impact on LVLS associations while additional adjustment for blood pressure and anti-hypertensive therapy had the largest impact on LVDSR associations (Additional file 1: Table S2).

**Table 3:** Cross-sectional associations of glucose and insulin measures with cardiac strain among CHS participants

Measure	Left ventricular longitudinal strain					
	Model 1^a^			Model 2^b^		
	N	Beta (95% CI)	p-value	N	Beta (95% CI)	p-value
Fasting glucose	2426	− 0.25 (− 0.39, − 0.12)	0.000	2426	− 0.07 (− 0.21, 0.07)	0.337
2 h glucose	2345	− 0.23 (− 0.37, − 0.10)	0.001	2345	− 0.04 (− 0.19, 0.10)	0.533
HOMA-IR	2426	− 0.24 (− 0.37, − 0.11)	0.000	2426	− 0.07 (− 0.20, 0.07)	0.337
Matsuda ISI	2336	0.41 (0.27, 0.54)	0.000	2336	0.18 (0.03, 0.33)	0.021
Stumvoll index	2336	− 0.21 (− 0.35, − 0.07)	0.002	2336	− 0.10 (− 0.24, 0.04)	0.178

Measure	Left ventricular early diastolic strain rate					
	Model 1			Model 2		
	N	Beta (95% CI)	p-value	N	Beta (95% CI)	p-value
Fasting glucose	2385	− 0.02 (− 0.03, − 0.01)	0.000	2385	− 0.01 (− 0.02, 0.00)	0.203
2 h glucose	2305	− 0.01 (− 0.02, − 0.01)	0.003	2305	− 0.00 (− 0.01, 0.01)	0.613
HOMA-IR	2385	− 0.02 (− 0.03, − 0.01)	0.000	2385	− 0.01 (− 0.02, 0.00)	0.232
Matsuda ISI	2296	0.03 (0.02, 0.04)	0.000	2296	0.01 (− 0.00, 0.02)	0.101
Stumvoll index	2296	− 0.01 (− 0.02, − 0.01)	0.002	2296	− 0.00 (− 0.01, 0.01)	0.455

Measure	Left atrial reservoir strain					
	Model 1			Model 2		
	N	Beta (95% CI)	p-value	N	Beta (95% CI)	p-value
Fasting glucose	2375	− 0.69 (− 1.28, − 0.09)	0.023	2375	− 0.19 (− 0.81, 0.43)	0.550
2 h glucose	2300	− 0.49 (− 1.10, 0.12)	0.113	2300	0.02 (− 0.62, 0.65)	0.959
HOMA-IR	2375	− 0.32 (− 0.91, 0.27)	0.292	2375	0.16 (− 0.46, 0.78)	0.610
Matsuda ISI	2291	0.89 (0.28, 1.50)	0.004	2291	0.21 (− 0.48, 0.89)	0.553
Stumvoll index	2291	− 0.42 (− 1.03, 0.19)	0.174	2291	− 0.06 (− 0.70, 0.58)	0.862

Mean ± SD for exposure and outcome variables: Fasting glucose, mg/dL = 102.77 + 19.24, 2-h glucose, mg/dL = 142.44 + 54.6, HOMA-IR = 3.77 + 4.46, Matsuda ISI = 3.64 + 2.23, Stumvoll index = 335.48 + 178.93, Left ventricular longitudinal strain, % = 14.80 + 3.54, Left ventricular early diastolic strain rate, s.^−1^ = 0.676 + 0.238, Left atrial reservoir strain, % = 42.14 + 15.22

Values shown are beta coefficients (95% confidence intervals) per SD increment in measure from linear regression models

*HOMA-IR* homeostatic model of insulin resistance, *ISI* insulin sensitivity index

^a^Model 1 adjusted for age, sex, race, site, speckle-tracking analyst, echo image and quality score

^b^Model 2 = Model 1 + waist circumference, smoking, alcohol, systolic blood pressure, antihypertensive therapy, LDL, HDL, eGFR, heart rate

**Table 4:** Effect of Model 2 covariates on glucose and insulin measure estimates (each covariate is added one at a time)

Left ventricular longitudinal strain									
Measure	Waist circumference	Systolic BP	BP medication	LDL cholesterol	HDL cholesterol	eGFR_cys_	Heart rate	Smoking	Alcohol
	Beta (95% CI)	Beta (95% CI)	Beta (95% CI)	Beta (95% CI)	Beta (95% CI)	Beta (95% CI)	Beta (95% CI)	Beta (95% CI)	Beta (95% CI)
	*p*-value	*p*-value	*p*-value	*p*-value	*p*-value	*p*-value	*p*-value	*p*-value	*p*-value
Fasting glucose	− 0.17 (− 0.31, − 0.03)	− 0.23 (− 0.37, − 0.09)	− 0.23 (− 0.37, − 0.10)	− 0.26 (− 0.39, − 0.12)	− 0.22 (− 0.36, − 0.08)	− 0.25 (− 0.39, − 0.12)	− 0.20 (− 0.33, − 0.06)	− 0.25 (− 0.39, − 0.12)	− 0.26 (− 0.39, − 0.12)
	0.018	0.001	0.001	< 0.001	0.002	< 0.001	0.004	< 0.001	< 0.001
2-h glucose	− 0.15 (− 0.29, − 0.01)	− 0.20 (− 0.34, − 0.06)	− 0.21 (− 0.35, − 0.07)	− 0.23 (− 0.37, − 0.10)	− 0.20 (− 0.34, − 0.06)	− 0.23 (− 0.37, − 0.09)	− 0.17 (− 0.31, − 0.03)	− 0.23 (− 0.37, − 0.10)	− 0.24 (− 0.38, − 0.10)
	0.031	0.004	0.003	0.001	0.005	0.001	0.017	0.001	0.001
HOMA-IR	− 0.14 (− 0.27, − 0.01)	− 0.21 (− 0.34, − 0.09)	− 0.21 (− 0.34, − 0.08)	− 0.23 (− 0.36, − 0.10)	0.20 (− 0.32, − 0.07)	− 0.20 (− 0.33, − 0.07)	− 0.19 (− 0.32, − 0.07)	− 0.23 (− 0.36, − 0.11)	− 0.23 (− 0.36, − 0.10)
	0.037	0.001	0.002	< 0.001	0.003	0.002	0.003	< 0.001	< 0.001
Matsuda ISI	0.29 (0.15, 0.44)	0.37 (0.23, 0.51)	0.38 (0.25, 0.52)	0.41 (0.27, 0.54)	0.36 (0.22, 0.50)	0.38 (0.25, 0.52)	0.35 (0.21, 0.48)	0.41 (0.28, 0.55)	0.41 (0.28, 0.55)
	< 0.001	< 0.001	< 0.001	< 0.001	< 0.001	< 0.001	< 0.001	< 0.001	< 0.001
Stumvoll index	− 0.11 (− 0.25, 0.03)	− 0.21 (− 0.35, − 0.07)	− 0.20 (− 0.33, − 0.06)	− 0.21 (− 0.35, − 0.07)	− 0.17 (− 0.31, − 0.03)	− 0.19 (− 0.33, − 0.05)	− 0.21 (− 0.35, − 0.08)	− 0.22 (− 0.36, − 0.08)	− 0.21 (− 0.35, − 0.08)
	0.122	0.003	0.006	0.002	0.016	0.008	0.002	0.002	0.002

Left ventricular early diastolic strain rate									
Measure	Waist circumference	Systolic BP	BP medication	LDL cholesterol	HDL cholesterol	eGFR_cys_	Heart rate	Smoking	Alcohol
	Beta (95% CI)	Beta (95% CI)	Beta (95% CI)	Beta (95% CI)	Beta (95% CI)	Beta (95% CI)	Beta (95% CI)	Beta (95% CI)	Beta (95% CI)
	*p*-value	*p*-value	*p*-value	*p*-value	*p*-value	*p*-value	*p*-value	*p*-value	*p*-value
Fasting glucose	− 0.01 (− 0.02, − 0.00)	− 0.02 (− 0.02, − 0.01)	− 0.02 (− 0.02, − 0.01)	− 0.02 (− 0.03, − 0.01)	− 0.01 (− 0.02, − 0.01)	− 0.02 (− 0.03, − 0.01)	− 0.02 (− 0.03, − 0.01)	− 0.02 (− 0.03, − 0.01)	− 0.02 (− 0.03, − 0.01)
	0.045	0.001	0.001	< 0.001	0.002	< 0.001	< 0.001	< 0.001	< 0.001
2-h glucose	− 0.01 (− 0.02, 0.00)	− 0.01 (− 0.02, − 0.00)	− 0.01 (− 0.02, − 0.00)	− 0.01 (− 0.02, − 0.00)	− 0.01 (− 0.02, − 0.00)	− 0.01 (− 0.02, − 0.00)	− 0.01 (− 0.02, − 0.00)	− 0.01 (− 0.02, − 0.00)	− 0.01 (− 0.02, − 0.00)
	0.146	0.015	0.012	0.003	0.012	0.004	0.006	0.003	0.003
HOMA-IR	− 0.01 (− 0.02, − 0.00)	− 0.02 (− 0.02, − 0.01)	− 0.01 (− 0.02, − 0.01)	− 0.02 (− 0.03, − 0.01)	− 0.01 (− 0.02, − 0.01)	− 0.01 (− 0.02, − 0.01)	− 0.02 (− 0.03, − 0.01)	− 0.02 (− 0.03, − 0.01)	− 0.02 (− 0.03, − 0.01)
	0.041	< 0.001	0.001	< 0.001	0.001	0.001	< 0.001	< 0.001	< 0.001
Matsuda ISI	0.01 (0.00, 0.02)	0.02 (0.01, 0.03)	0.02 (0.01, 0.03)	0.03 (0.02, 0.04)	0.02 (0.01, 0.03)	0.02 (0.01, 0.03)	0.03 (0.02, 0.03)	0.03 (0.02, 0.04)	0.03 (0.02, 0.04)
	0.004	< 0.001	< 0.001	< 0.001	< 0.001	< 0.001	< 0.001	< 0.001	< 0.001
Stumvoll index	− 0.01 (− 0.02, 0.00)	− 0.01 (− 0.02, − 0.00)	− 0.01 (− 0.02, − 0.00)	− 0.01 (− 0.02, − 0.01)	− 0.01 (− 0.02, − 0.00)	− 0.01 (− 0.02, − 0.00)	− 0.01 (− 0.02, − 0.01)	− 0.01 (− 0.02, − 0.01)	− 0.01 (− 0.02, − 0.00)
	0.268	0.003	0.009	0.003	0.017	0.014	0.002	0.002	0.003

Left atrial reservoir strain									
Measure	Waist circumference	Systolic BP	BP medication	LDL cholesterol	HDL cholesterol	eGFR_cys_	Heart rate	Smoking	Alcohol
	Beta (95% CI)	Beta (95% CI)	Beta (95% CI)	Beta (95% CI)	Beta (95% CI)	Beta (95% CI)	Beta (95% CI)	Beta (95% CI)	Beta (95% CI)
	*p*-value	*p*-value	*p*-value	*p*-value	*p*-value	*p*-value	*p*-value	*p*-value	*p*-value
Fasting glucose	− 0.40 (− 1.01, 0.22)	− 0.61 (− 1.21, 0.00)	− 0.64 (− 1.24, − 0.03)	− 0.65 (− 1.26, − 0.05	− 0.62 (− 1.23, − 0.01)	− 0.68 (− 1.28, − 0.07)	− 0.59 (− 1.20, 0.02)	− 0.68 (− 1.29, − 0.08)	− 0.67 (− 1.28, − 0.07)
	0.210	0.049	0.040	0.034	0.047	0.028	0.056	0.027	0.029
2-h glucose	− 0.19 (− 0.82, 0.43)	− 0.39 (− 1.01, 0.23)	− 0.44 (− 1.06, 0.18)	− 0.46 (− 1.08, 0.15)	− 0.41 (− 1.03, 0.21)	− 0.47 (− 1.08, 0.14)	− 0.37 (− 0.99, 0.25)	− 0.49 (− 1.10, 0.13)	− 0.47 (− 1.08, 0.15)
	0.543	0.219	0.163	0.138	0.190	0.134	0.240	0.120	0.138
HOMA-IR	− 0.01 (− 0.60, 0.57)	− 0.27 (− 0.84, 0.29)	− 0.26 (− 0.84, 0.31)	− 0.33 (− 0.89, 0.24)	− 0.26 (− 0.83, 0.31)	− 0.28 (− 0.85, 0.29)	− 0.26 (− 0.83, 0.31)	− 0.33 (− 0.89, 0.24)	− 0.31 (− 0.88, 0.26)
	0.965	0.343	0.366	0.257	0.375	0.333	0.367	0.259	0.283
Matsuda ISI	0.41 (− 0.24, 1.07)	0.75 (0.13, 1.38)	0.82 (0.19, 1.44)	0.82 (0.20, 1.44)	0.77 (0.14, 1.41)	0.83 (0.21, 1.46)	0.76 (0.14, 1.37)	0.87 (0.25, 1.48)	0.84 (0.23, 1.46)
	0.218	0.017	0.010	0.009	0.017	0.009	0.017	0.006	0.007
Stumvoll index	− 0.04 (− 0.67, 0.60)	− 0.38 (− 0.99, 0.23)	− 0.36 (− 0.97, 0.26)	− 0.38 (− 0.99, 0.23)	− 0.31 (− 0.93, 0.32)	− 0.36 (− 0.98, 0.26)	− 0.38 (− 1.00, 0.23)	− 0.39 (− 1.00, 0.23)	− 0.38 (− 1.00, 0.23)
	0.910	0.222	0.255	0.224	0.337	0.256	0.219	0.218	0.223

Beta-coefficients above already adjusted for Model 1 covariates—age, sex, race, site, speckle-tracking analyst, echo image and quality score

*BP* blood pressure, *LDL* low-density lipoprotein, *HDL* high-density lipoprotein, *eGFR*_*cys*_ estimated glomerular filtration rate cystatin C, *HOMA-IR* homeostatic model of insulin resistance, *ISI* insulin sensitivity index

When we tested glycemic measures as potential mediators of the association of waist circumference with each of the cardiac strain measures, they mediated variable but small degrees of the observed associations. For LVLS, only two-hour glucose had any measurable degree of mediation. Waist circumference was associated with an increment of − 0.0351 per SD (p = 0.02), of which 14% was mediated by two-hour glucose. For LV EDSR, HOMA-IR, Matsuda, and Stumvoll all tended to provide similar degrees of mediation. Waist circumference was associated with an increment of − 0.003 per SD (p = 0.07–0.004, depending on the model), of which 11–18% was mediated by any one of these measures. For LALS, fasting glucose and HOMA-IR provided similar degrees of mediation. Waist circumference was associated with an increment of − 0.15335 per SD (p = 0.02), of which 17–21% was mediated by either of these two closely related measures.

## Discussion

Among older adults, higher insulin sensitivity, as measured by the Matsuda ISI, was independently associated with better adjusted LVLS. All other observed associations for glucose and insulin measures with each of the 3 cardiac strain measures were significantly attenuated after adjustment for cardiovascular risk factors, and this was particularly driven by central adiposity.

The association between fasting glucose measures and adverse cardiac mechanics has been previously evaluated in population-based cohorts free of baseline cardiovascular disease but these comprised younger individuals and mainly focused on LVLS measures [14–16, 26]. The mean participant age for these studies was between 50 and 60 years old. Both the Coronary Artery Risk Development in Young Adult (CARDIA) and Framingham Heart Study (FHS) Offspring and 3rd generation cohorts, 3179 and 6231 participants respectively, found that higher HOMA-IR was associated with worse LVLS, a finding that persisted after adjustment for waist circumference [14, 15]. Similar results were observed in a smaller study of 539 Japanese participants [26]. Additionally, the CARDIA study also found that higher HOMA-IR was also associated with worse LVDSR [14]. Finally, in a study of 627 Flemish individuals, higher HOMA-IR was associated with a greater decline in LVLS over an almost 5-year period [16]. The current study is the first to report a comprehensive assessment of the relationship for both fasting and post-glucose load measures with a variety of subclinical cardiac dysfunction measures in an older population.

It is unclear why, in contrast to the CARDIA and FHS studies mentioned above, HOMA-IR was also not associated with LVLS in CHS. The impact of waist circumference in this older age group may be potentially larger than that seen in CARDIA and FHS; however, further study is needed. The observed differences found between fasting glucose post-load glucose measures in this study also highlight the fact that IR involves multiple metabolic tissues, skeletal muscle, liver, and adipose tissue, and measurement depends on the tissue type. These two measures represent different insulin sensitivity and release patterns and may capture IR in certain metabolic tissue types better than others [27]. Fasting IR is a central measure that assesses hepatic tissue glucose uptake in response to insulin while post-glucose load IR is a peripheral measure that mainly measures skeletal muscle and, to a lesser extent, adipose tissue glucose uptake in response to insulin. Given that skeletal muscle and adipose tissue IR are important determinants of increased free fatty acid levels and ectopic fat deposition, it was important to assess their potential effects separately from those of hepatic IR [28]. Our findings suggest that peripheral IR may be more important than central IR with respect to the presence of subclinical cardiac dysfunction in an older population, much as post-load measures are more strongly associated with prognosis in this population.

Higher levels of inflammation, activation of renin angiotensin system, and impaired coronary flow reserve have all been observed in individuals with increased insulin resistance and these may all potentially have an underlying role in the association between Matsuda ISI and subclinical LV dysfunction seen here [29–34]. Insulin resistance itself has also been shown to have a direct negative effect on myocardial mechanics through decreased mechano-energetic efficiency [35]. It may also promote activation of nutrient sensing pathways and overexpression of the Sodium-Hydrogen exchanger in cardiomyocytes, resulting in increased oxidative stress, cardiomyocyte injury, and systolic dysfunction [36]. Myocardial glucose transporter expression is reduced in insulin resistance and the resulting shift towards fatty acid metabolism exacerbates oxygen demands, increases oxidative stress, and impairs cardiomyocyte calcium handling [37, 38]. The excessive free fatty acid can also enhance cardiomyocyte apoptosis via production of lipotoxic intermediates [38, 39].

The overall lack of an association, however, between all other glycemic and insulin measures and cardiac strain after adjusting for waist circumference suggests that much of the higher risk is, in fact, related to the presence of visceral adiposity. Prior CHS work has already demonstrated that it is particularly strongly associated with measures of subclinical cardiac dysfunction [24]. The unadjusted associations observed for glycemic and insulin measures may have reflected the metabolic and humoral changes induced by visceral adiposity. Central obesity can trigger various endocrine, inflammatory, and neuronal pathways that ultimately result in IR [40–42]. Obesity is associated with increased fatty acid production, altered adipokine secretion, and increased adipocyte production of inflammatory cytokines, all of which can inhibit insulin signaling through modification of various intracellular pathways [42]. In addition, obesity can alter the central response to hormonal and nutrient signals and alter peripheral insulin sensitivity [42]. Thus, our findings support the role of fat accumulation in the metabolic dysregulation of aging and its cardiovascular consequences. [43]

Our study has limitations. Correlations of fasting and 2-h measures of IR with directly measured IR are only moderate; however, invasive glycemic clamp testing places a large burden on research participants and is impractical in epidemiologic study [44]. Speckle-tracking was performed retrospectively on echocardiograms that were acquired without specific attention to optimizing endocardial border definition. However, the majority of images acquired were of at least adequate quality, and image quality was entered into all regression analyses as a covariate. Data was not collected for additional LV strain measures, including radial and circumferential strain. The population is older, mainly white, and, consistent with differences in mortality, disproportionately female, so we cannot necessarily generalize to other populations. Repeated, comparable cardiac strain measures are not available in CHS, and therefore we could not determine associations of glucose dysregulation with longitudinal changes in cardiac strain. Finally, the clinical significance of reported associations is limited by the fact that it remains unknown whether targeted efforts to improve LV cardiac mechanics is effective in disease prevention.

In a community-dwelling population of older adults, while a higher post-glucose load insulin sensitivity was independently associated better LVLS, nearly all observed associations for glucose and insulin measures with each of the 3 cardiac strain measures were significantly attenuated after adjustment for central adiposity. Our findings suggest that the maladaptive alterations in glucose and NEFA release and uptake induced by visceral adiposity, along with the consequences of attendant production of bioactive factors, are more related to subclinical cardiac dysfunction than the actual presence of hyperglycemia or insulin resistance itself. Further study is needed to better discern mechanistic pathways that describe the interaction of visceral adiposity, hyperglycemia, and insulin resistance and how it can lead to subclinical cardiac dysfunction.

## Supplementary Information

**Additional file 1**: **Table S1.** Baseline characteristics of CHS participants according to 2-h glucose quartiles*. **Table S2.** Effect of covariates on glucose and insulin measure estimates after including waist circumference in Model 1 (each covariate is added one at a time)*.

## Data Availability

The CHS data are available to qualifying investigators directly from the study by following CHS policies and procedures (https://chs-nhlbi.org/CHS_DistribPolicy), and also through dbGaP (http://www.ncbi.nlm.nih.gov/gap) and BioLINCC (https://biolincc.nhlbi.nih.gov). A variety of stored biospecimens are also available from the study, including DNA, serum, plasma, and urine.
