# Supplementary material for: Glucose dysregulation and subclinical cardiac dysfunction in older adults: The Cardiovascular Health Study
Source: Cardiovasc Diabetol. 2022 Jun 20;21:112. doi: 10.1186/s12933-022-01547-z (PMC9210635; doi:10.1186/s12933-022-01547-z)
Supplement: Supplementary file 1 — Additional file 1: Table S1. Baseline characteristics of CHS participants according to 2-h glucose quartiles*. Table S2. Effect of covariates on glucose and insulin measure estimates after including waist circumference in Model 1 (each covariate is added one at a time)*. [file 12933_2022_1547_MOESM1_ESM.docx]

Additional file 1

**Table S1.** Baseline characteristics of CHS participants according to 2-hour glucose quartiles^*^

| Characteristic | Post-load glucose quartile | | | |
| --- | --- | --- | --- | --- |
|  | <=107 mg/dl | >107-132 mg/dl | >132-163 mg/dl | >163 mg/dl |
| Age, years | 71.46 ± 4.99 | 71.53 ± 4.91 | 72.17 ± 5.13 | 72.82 ± 5.36 |
| Waist circumference, cm | 88.56 ± 12.67 | 90.52 ± 11.79 | 92.82 ± 12.73 | 95.96 ± 12.87 |
| Heart rate | 32.96 ± 4.57 | 33.20 ± 4.90 | 33.84 ± 5.12 | 34.62 ± 5.66 |
| Physical activity, kcal/week | 1954.58 ± 2071.04 | 2015.23 ± 2118.21 | 1966.69 ± 2252.08 | 1692.43 ± 2058.29 |
| Systolic BP, mmHg | 129.51 ± 21.36 | 134.80 ± 19.88 | 135.71 ± 20.38 | 138.67 ± 21.15 |
| LDL, mg/dl | 129.02 ± 34.52 | 132.72 ± 34.46 | 133.26 ± 34.67 | 133.13 ± 38.18 |
| HDL, mg/dl | 59.47 ± 16.01 | 57.45 ± 16.01 | 55.52 ± 15.19 | 54.47 ± 15.27 |
| eGFR-cys_c_ | 82.11 ± 17.83 | 80.74 ± 17.88 | 78.25 ± 17.67 | 77.85 ± 20.75 |
| Male | 205 (34.3%) | 214 (35.7%) | 184 (32.2%) | 200 (34.4%) |
| Black | 19 (3.2%) | 26 (4.3%) | 16 (2.8%) | 24 (4.1%) |
| Hypertension | 161 (26.9%) | 207 (34.6%) | 217 (37.9%) | 299 (51.4%) |
| Stroke | 12 (2.0%) | 12 (2.0%) | 10 (1.7%) | 25 (4.3%) |
| Anti-hypertensive use | 166 (27.8%) | 194 (32.3%) | 198 (34.6%) | 283 (48.6%) |
| Smoking status |  |  |  |  |
| Never | 254 (42.5%) | 278 (46.3%) | 287 (50.2%) | 300 (51.5%) |
| Former | 257 (43.0%) | 249 (41.5%) | 221 (38.6%) | 222 (38.1%) |
| Current | 87 (14.5%) | 73 (12.2%) | 64 (11.2%) | 60 (10.3%) |
| Alcohol use, drinks/wk |  |  |  |  |
| None | 237 (39.6%) | 241 (40.2%) | 283 (49.5%) | 289 (49.7%) |
| 1-7 | 262 (43.8%) | 267 (44.5%) | 220 (38.5%) | 211 (36.3%) |
| >7 | 99 (16.6%) | 92 (15.3%) | 69 (12.1%) | 82 (14.1%) |

LDL=low-density lipoprotein, HDL=high-density lipoprotein, eGFR_cys_=estimated glomerular filtration rate cystatin C

*Continuous variables are expressed as mean (SD). Categorical variables are N (percent).

**Table S2.** Effect of covariates on glucose and insulin measure estimates after including waist circumference in Model 1

(each covariate is added one at a time)^*^

| **Left ventricular longitudinal strain** | | | | | | | | | |
| --- | --- | --- | --- | --- | --- | --- | --- | --- | --- |
|  | | | | | | | | | |
| **Measure** | Model 1 | Systolic BP | BP medication | LDL cholesterol | HDL cholesterol | eGFR_cys_ | Heart rate | Smoking | Alcohol |
|  | Beta  (95% CI) | Beta  (95% CI) | Beta  (95% CI) | Beta  (95% CI) | Beta  (95% CI) | Beta  (95% CI) | Beta  (95% CI) | Beta  (95% CI) | Beta  (95% CI) |
|  | *p*-value | *p*-value | *p*-value | *p*-value | *p*-value | *p*-value | *p*-value | *p*-value | *p*-value |
|  | | | | | | | | | |
| Fasting glucose | 0.17  (-0.30, -0.03) | -0.15  (-0.29, -0.01) | -0.15  (-0.29, -0.02) | -0.17  (-0.30, -0.03) | -0.15  (-0.29, -0.01) | -0.17  (-0.31, -0.03) | -0.11  (-0.24, 0.03) | -0.16  (-0.30, -0.02) | -0.17  (-0.31, -0.03) |
|  | 0.018 | 0.035 | 0.029 | 0.017 | 0.031 | 0.015 | 0.122 | 0.022 | 0.017 |
| 2-hour glucose | -0.15  (-0.29, -0.01) | -0.13  (-0.27, 0.01) | -0.14  (-0.28, 0.00) | -0.15  (-0.29, -0.01) | -0.14  (-0.28, 0.00) | -0.15  (-0.29, -0.01) | -0.08  (-0.22, 0.05) | -0.15  (-0.29, -0.01) | -0.16  (-0.30, -0.02) |
|  | 0.031 | 0.072 | 0.050 | 0.031 | 0.047 | 0.031 | 0.233 | 0.035 | 0.026 |
| HOMA-IR | -0.15  (-0.28, -0.01) | -0.14 (-0.27, 0.00) | -0.13 (-0.27, 0.01) | -0.15 (-0.28, -0.01) | -0.13 (-0.27, 0.00) | -0.13 (-0.27, 0.01) | -0.11 (-0.24, 0.03) | -0.15 (-0.28, -0.01) | -0.15 (-0.29, -0.01) |
|  | 0.037 | 0.053 | 0.067 | 0.038 | 0.059 | 0.064 | 0.125 | 0.035 | 0.034 |
| Matsuda ISI | 0.29  (0.15, 0.44) | 0.27  (0.12, 0.41) | 0.28  (0.13, 0.43) | 0.30  (0.15, 0.44) | 0.28  (0.13, 0.42) | 0.28  (0.14, 0.43) | 0.23  (0.08, 0.38) | 0.30  (0.15, 0.45) | 0.30  (0.15, 045) |
|  | <0.001 | <0.001 | <0.001 | <0.001 | <0.001 | <0.001 | 0.002 | <0.001 | <0.001 |
| Stumvoll index | -0.11  (-0.25, 0.03) | -0.11  (-0.26, 0.03) | -0.10  (-0.24, 0.04) | -0.11  (-0.25, 0.03) | -0.10  (-0.24, 0.05) | -0.10  (-0.24, 0.04) | -0.11  (-0.25, 0.03) | -0.12  (-0.26, 0.02) | -0.11  (-0.25, 0.03) |
|  | 0.122 | 0.111 | 0.156 | 0.123 | 0.186 | 0.179 | 0.108 | 0.099 | 0.119 |
|  | | | | | | | | | |
| **Left ventricular early diastolic strain rate** | | | | | | | | | |
|  | | | | | | | | | |
| **Measure** | Model 1 | Systolic BP | BP medication | LDL cholesterol | HDL cholesterol | eGFR_cys_ | Heart rate | Smoking | Alcohol |
|  | Beta  (95% CI) | Beta  (95% CI) | Beta  (95% CI) | Beta  (95% CI) | Beta  (95% CI) | Beta  (95% CI) | Beta  (95% CI) | Beta  (95% CI) | Beta  (95% CI) |
|  | *p*-value | *p*-value | *p*-value | *p*-value | *p*-value | *p*-value | *p*-value | *p*-value | *p*-value |
|  | | | | | | | | | |
| Fasting glucose | -0.01  (-0.02, -0.00) | -0.01  (-0.02, 0.00) | -0.01  (-0.02, 0.00) | -0.01  (-0.02, 0.00) | -0.01  (-0.02, 0.00) | -0.01  (-0.02, 0.00) | -0.01  (-0.02, 0.00) | -0.01  (-0.02, 0.00) | -0.01  (-0.02, 0.00) |
|  | 0.045 | 0.097 | 0.090 | 0.044 | 0.062 | 0.036 | 0.069 | 0.050 | 0.043 |
| 2-hour glucose | -0.01  (-0.02, 0.00) | 0.00  (-0.01, 0.00) | -0.01  (-0.02, 0.00) | -0.01  (-0.02, 0.00) | -0.01  (-0.02, 0.00) | -0.01  (-0.02, 0.00) | -0.01  (-0.02, 0.00) | -0.01  (-0.02, 0.00) | -0.01  (-0.02, 0.00) |
|  | 0.146 | 0.314 | 0.255 | 0.147 | 0.185 | 0.146 | 0.222 | 0.161 | 0.146 |
| HOMA-IR | -0.01  (-0.02, -0.00) | -0.01  (-0.02, 0.00) | -0.01  (-0.02, 0.00) | -0.01  (-0.02, 0.00) | -0.01  (-0.02, 0.00) | -0.01  (-0.02, 0.00) | -0.01  (-0.02, 0.00) | -0.01  (-0.02, 0.00) | -0.01  (-0.02, 0.00) |
|  | 0.041 | 0.063 | 0.104 | 0.041 | 0.054 | 0.084 | 0.054 | 0.038 | 0.041 |
| Matsuda ISI | 0.01  (0.00, 0.02) | 0.01  (0.00, 0.02) | 0.01  (0.00, 0.02) | 0.01  (0.00, 0.02) | 0.01  (0.00, 0.02) | 0.01  (0.00, 0.02) | 0.01  (0.00, 0.02) | 0.01  (0.00, 0.02) | 0.01  (0.00, 0.02) |
|  | 0.004 | 0.017 | 0.013 | 0.004 | 0.008 | 0.009 | 0.007 | 0.004 | 0.004 |
| Stumvoll index | -0.01  (-0.02, 0.00) | -0.01  (-0.02, 0.00) | 0.00  (-0.01, 0.01) | -0.01  (-0.02, 0.00) | 0.00  (-0.01, 0.01) | 0.00  (-0.01, 0.01) | -0.01  (-0.02, 0.00) | -0.01  (-0.02, 0.00) | -0.01  (-0.01, 0.00) |
|  | 0.268 | 0.245 | 0.370 | 0.268 | 0.350 | 0.420 | 0.258 | 0.245 | 0.282 |
|  | | | | | | | | | |
| **Left Atrial Reservoir Strain** | | | | | | | | | |
|  | | | | | | | | | |
| **Measure** | Model 1 | Systolic BP | BP medication | LDL cholesterol | HDL cholesterol | eGFR_cys_ | Heart rate | Smoking | Alcohol |
|  | Beta  (95% CI) | Beta  (95% CI) | Beta  (95% CI) | Beta  (95% CI) | Beta  (95% CI) | Beta  (95% CI) | Beta  (95% CI) | Beta  (95% CI) | Beta  (95% CI) |
|  | *p*-value | *p*-value | *p*-value | *p*-value | *p*-value | *p*-value | *p*-value | *p*-value | *p*-value |
|  | | | | | | | | | |
| Fasting glucose | -0.40  (-1.01, 0.22) | -0.34  (-0.95, 0.28) | -0.37  (-0.98, 0.25) | -0.38  (-0.99, 0.24) | -0.38  (-1.00, 0.23) | -0.39  (-1.01, 0.22) | -0.30  (-0.91, 0.32) | -0.39  (-1.00, 0.22) | -0.39  (-1.00, 0.22) |
|  | 0.210 | 0.281 | 0.241 | 0.228 | 0.221 | 0.206 | 0.346 | 0.215 | 0.214 |
| 2-hour glucose | -0.19  (-0.82, 0.43) | -0.12  (-0.75, 0.50) | -0.18  (-0.80, 0.45) | -0.19  (-0.81, 0.43) | -0.18  (-0.81, 0.44) | -0.19  (-0.81, 0.43) | -0.08  (-0.70, 0.54) | -0.20  (-0.82, 0.42) | -0.19  (-0.81, 0.43) |
|  | 0.543 | 0.701 | 0.581 | 0.552 | 0.564 | 0.543 | 0.802 | 0.530 | 0.555 |
| HOMA-IR | -0.01  (-0.60, 0.57) | 0.02  (-0.59, 0.62) | 0.02  (-0.59, 0.63) | -0.03  (-0.63, 0.58) | 0.00  (-0.61, 0.61) | 0.00  (-0.61, 0.61) | 0.05  (-0.55, 0.66) | -0.01  (-0.62, 0.59) | -0.01  (-0.62, 0.60) |
|  | 0.965 | 0.956 | 0.943 | 0.930 | 0.991 | 0.998 | 0.860 | 0.963 | 0.980 |
| Matsuda ISI | 0.41  (-0.24, 1.07) | 0.33  (-0.33, 1.00) | 0.40  (-0.27, 1.06) | 0.40  (-0.26, 1.06) | 0.40  (-0.26, 1.07) | 0.42  (-0.25, 1.08) | 0.30  (-0.36, 0.96) | 0.43  (-0.23, 1.09) | 0.41  (-0.25, 1.07) |
|  | 0.218 | 0.323 | 0.243 | 0.238 | 0.236 | 0.218 | 0.373 | 0.200 | 0.222 |
| Stumvoll index | -0.04  (-0.67, 0.60) | -0.05  (-0.68, 0.58) | -0.03  (-0.66, 0.61) | -0.04  (-0.67, 0.59) | -0.02  (-0.66, 0.61) | -0.03  (-0.67, 0.60) | -0.04  (-0.67, 0.59) | -0.04  (-0.67, 0.60) | -0.04  (-0.67, 0.60) |
|  | 0.910 | 0.880 | 0.938 | 0.902 | 0.944 | 0.915 | 0.910 | 0.911 | 0.913 |

BP=blood pressure, LDL=low-density lipoprotein, HDL=high-density lipoprotein, eGFR_cys_=estimated glomerular filtration rate cystatin C, HOMA-IR= homeostatic model of insulin resistance, ISI=insulin sensitivity index

*Beta-coefficients above already adjusted for Model 1 covariates—age, sex, race, site, speckle-tracking analyst, echo image and quality score, and waist circumference.
